# Supplementary material for: Communicating Two States in Perovskite Revealed by Time-Resolved Photoluminescence Spectroscopy
Source: Sci Rep. 2018 Nov 7;8:16482. doi: 10.1038/s41598-018-34645-8 (PMC6220243; doi:10.1038/s41598-018-34645-8)
Supplement: Supplementary file 1 — Supplementary Information [file 41598_2018_34645_MOESM1_ESM.docx]

**Supplementary Information**

**Communicating Two-States in Perovskite Revealed by Time-Resolved Photoluminescence Spectroscopy**

Yanwen Chen^#1^, Tianmeng Wang^#1^, Zhipeng Li^1,2^, Huanbin Li^1,3^, Tao Ye^3^, Christine Wetzel^4^, Hanying Li^3,*^, Su-Fei Shi^1,5,*^

^1^ The Department of Chemical and Biological Engineering, Rensselaer Polytechnic Institute, Troy, NY 12180

^2^ School of Chemistry and Chemical Engineering, Shanghai Jiao Tong University, Shanghai, 200240, China

^3^ MOE Key Laboratory of Macromolecule Synthesis and Functionalization, State Key Laboratory of Silicon Materials, Department of Polymer Science and Engineering, Zhejiang University, Hangzhou, 310027, P. R. China.

^4^ The Department of Physics, Applied Physics and Astronomy, Rensselaer Polytechnic Institute, Troy, NY 12180

^5^ The Department of Electrical, Computer and Systems Engineering, Rensselaer Polytechnic Institute, Troy, NY 12180

^#^ These authors contributed equally to this work

*Corresponding author: [hanying_li@zju.edu.cn](mailto:hanying_li@zju.edu.cn), [shis2@rpi.edu](mailto:shis2@rpi.edu)

**This PDF file includes:**

- Materials and Methods
- Figure S1. Scanning electron microscopy image of the MAPbI_3_ thin film.
- Figure S2. Schematic of the photoluminescence (PL) and time-resolved photoluminescence (TRPL) spectroscopy setup.
- Figure S3. PL measurement of the MAPbI_3_ thin film at the room temperature (RT).
- Figure S4. PL measurement of the MaPbI_3_ thin film at various spots.
- Figure S5. Fluence-dependent TRPL fitting of MAPbI_3_ thin film at 77 K.
- Figure S6. Temperature dependence of the PL spectra.
- Figure S7. Excitation wavelength dependent TRPL of MAPBI3 thin film at RT.

**Section S1. Materials and Methods**

1. **Sample Preparation**

The MAPbI_3_ powder was synthesized and dissolved at a concentration of 1 mol/L in dimethylformamide (DMF). Microscope slides were washed sequentially with soap, de-ionized water, acetone, and isopropanol before they were finally treated under oxygen plasma for 20 minutes to remove the organic residues. The MAPbI_3_ solution was spin-coated at 3000 rpm for 60 seconds, and the substrates were subsequently heated at 100 ^o^C on a hotplate in the glove box for 10 minutes to improve film quality. The morphology of MAPbI_3_ thin film is shown in Figure S1.


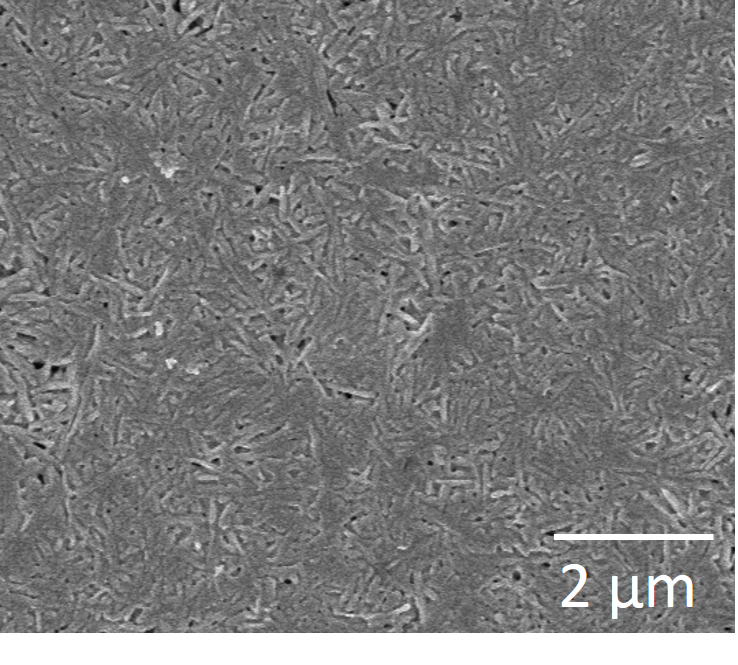


**Figure S1. Scanning electron microscopy image of the MAPbI_3_ thin film.**

1. **Optical Spectroscopy**

The steady-state photoluminescence (PL) and time-resolved photoluminescence (TRPL) spectroscopy measurements were performed with a home-built confocal microscope setup with either a CW or a femtosecond pulsed laser (repetition rate: 80 MHz). The excitation power of CW laser was typically maintained below 100 μW to prevent any sample degradation.

The TRPL measurement was performed by a Time-Correlated Single Photon Counting (TCSPC) module (PicoQuant TimeHarp-260) combined with an Avalanche Photo-Diode (MPD SPAD) through a spectrograph.

The samples were kept in vacuum (${<10}^{-6}$ Torr) during the measurements.

**
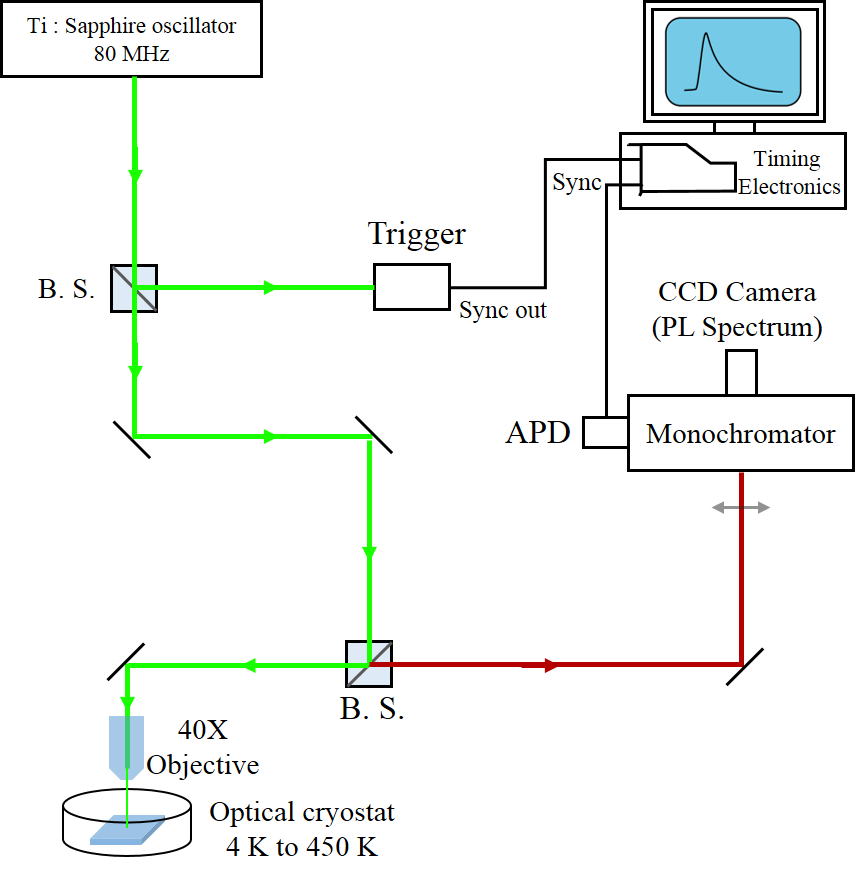
**

**Figure S2. Schematic of the photoluminescence (PL) and time-resolved photo-luminescence (TRPL) spectroscopy setup.**

**Section S2. Excitation Power Dependent PL at Room Temperature (RT)**

We characterized the photoluminescence (PL) spectra of the thin film at room temperature, using a continuous wave (CW) laser excitation centered at 2.33 eV ($\lambda$ = 532 nm). The MAPbI_3_ film at RT only exhibits a single emission peak centered at 1.61 eV. Figure S3a shows the PL spectra under different excitation powers, and the integrated PL intensity can be fitted using a power law as $I \sim P^{\alpha}$ with$\alpha=1.13$ (Fig. S3b). We also investigated the TRPL with different excitation fluences (Fig. S3c) for pulsed laser excitation, and the lifetime of the excited carriers (obtained by a mono-exponential fitting) decreases with the increasing fluence (Fig. S3d).


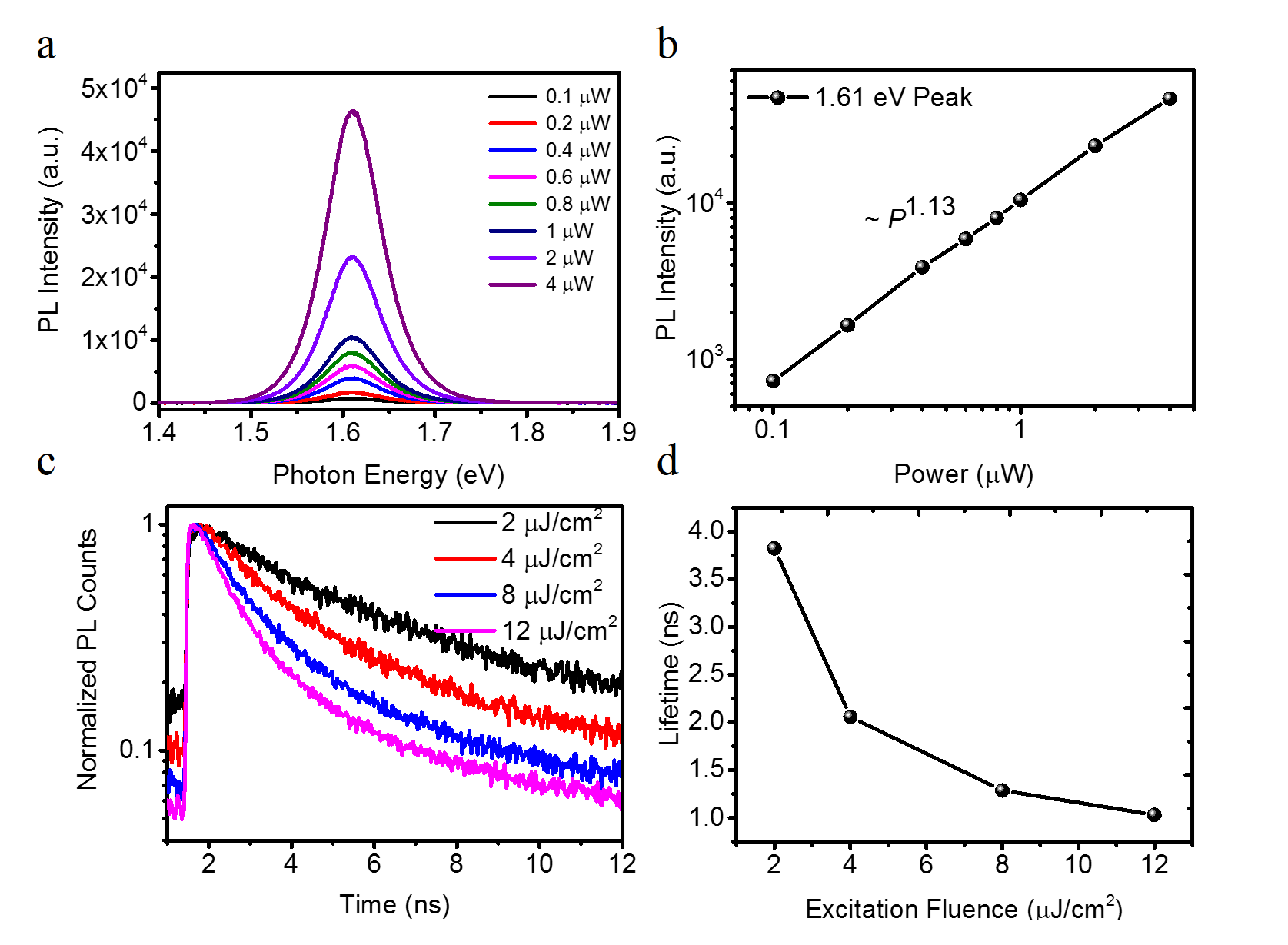


**Figure S3. PL measurement of the MAPbI_3_ thin film at RT.** (a) The power dependence of the steady-state PL spectra. (b) Logarithm plot of the PL intensity versus excitation power. The data show a power-law dependence with α = 1.13. (c) Normalized TRPL spectra as a function of the excitation fluence. (d) Fluence dependent lifetime obtained by a mono-exponential fitting.

We also measured the PL spectra at multiple positions (as shown in Fig. S4). Although the PL intensity may vary at different positions, the two emission peaks in PL spectra are universal at 77K.


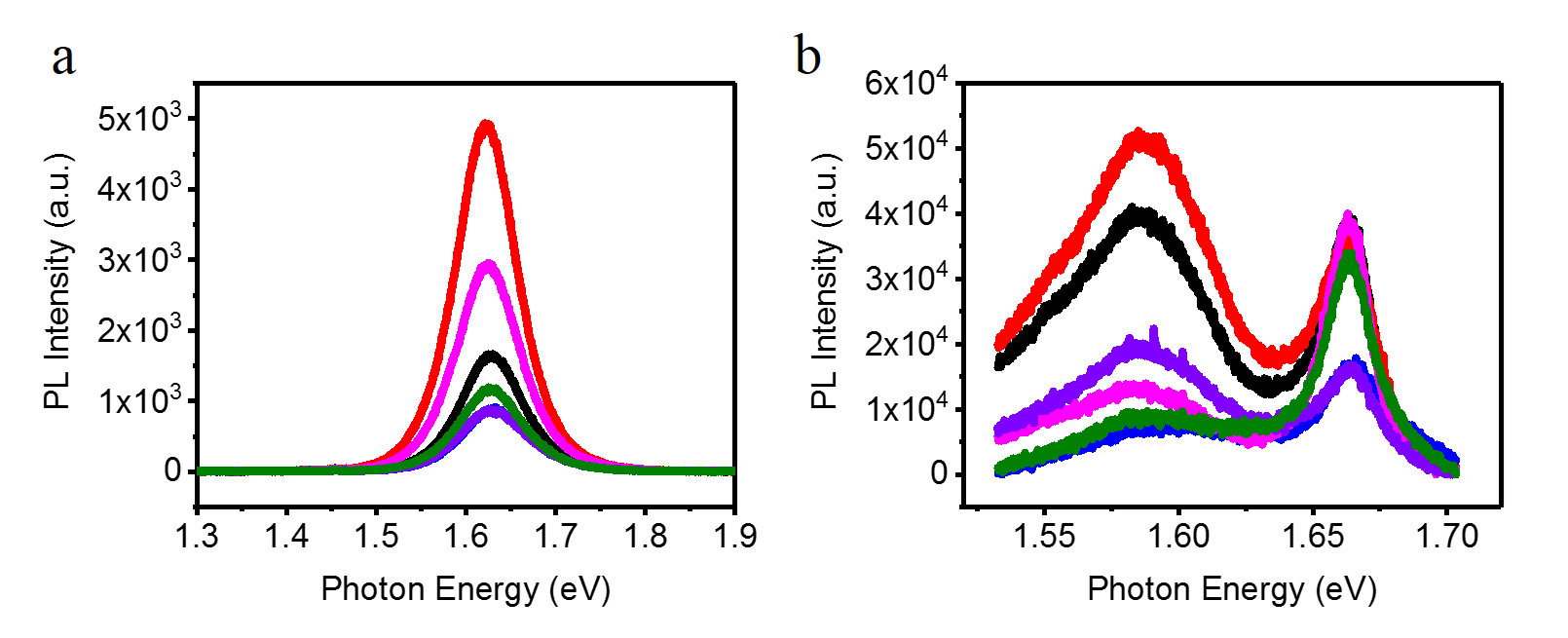


**Figure S4. PL spectra of various spots on MAPBI_3_ thin film at RT (a) and 77K (b) with the CW laser excitation centered at 2.33 eV (λ = 532 nm). The excitation power is 1µW.**

**Section S3. Two-level system modeling**

We use a two-level system model to describe the communication process between the HE and LE states, which is given by the rate equations:

$\frac{dn_{1}}{dt}=-k_{1}n_{1}-k_{12}n_{1}(\frac{{N_{0}-n}_{2}}{N_{0}})$ (1)

$\frac{dn_{2}}{dt}=-k_{2}n_{2}+k_{12}n_{1}(\frac{{N_{0}-n}_{2}}{N_{0}})$ (2)

where n_1_ and n_2_ are the optically excited carriers at the HE and LE state, respectively. k_1_ is the decay rate for HE state, k_2_ is the decay rate for the LE state, and k_12_ is the injection rate of carriers from the HE to LE state. N_0_ is the maximum state that can be occupied in the LE state.

We then fit our experimental data with the model using four fitting parameters, namely k_1_, k_2_, k_12_, and N_0._ Since the power law of both the HE and LE states is close to 1 (See Fig. 1d), we can directly use the TRPL counts rather than the carriers density for the fitting. Hence one may conclude that a reasonable starting value for N_0_ would be the maximum counts of the LE state TRPL decay curves. The initial values for k_1_ and k_2_ could be obtained from mono-exponential decay fitting, and the injection rate k_12_ could be estimated by the rising time in LE state TRPL curves.

Using these initial values, we performed the fitting of the PL intensity decay curves to rectify the parameters values, which then was used to calculate the TRPL counts again. This iteration procedure was repeated until satisfactory agreement between the experimental TRPL decay and the fitting is achieved. And the fittings to the TRPL decays under several different excitation fluences is shown in Fig. S5.

**
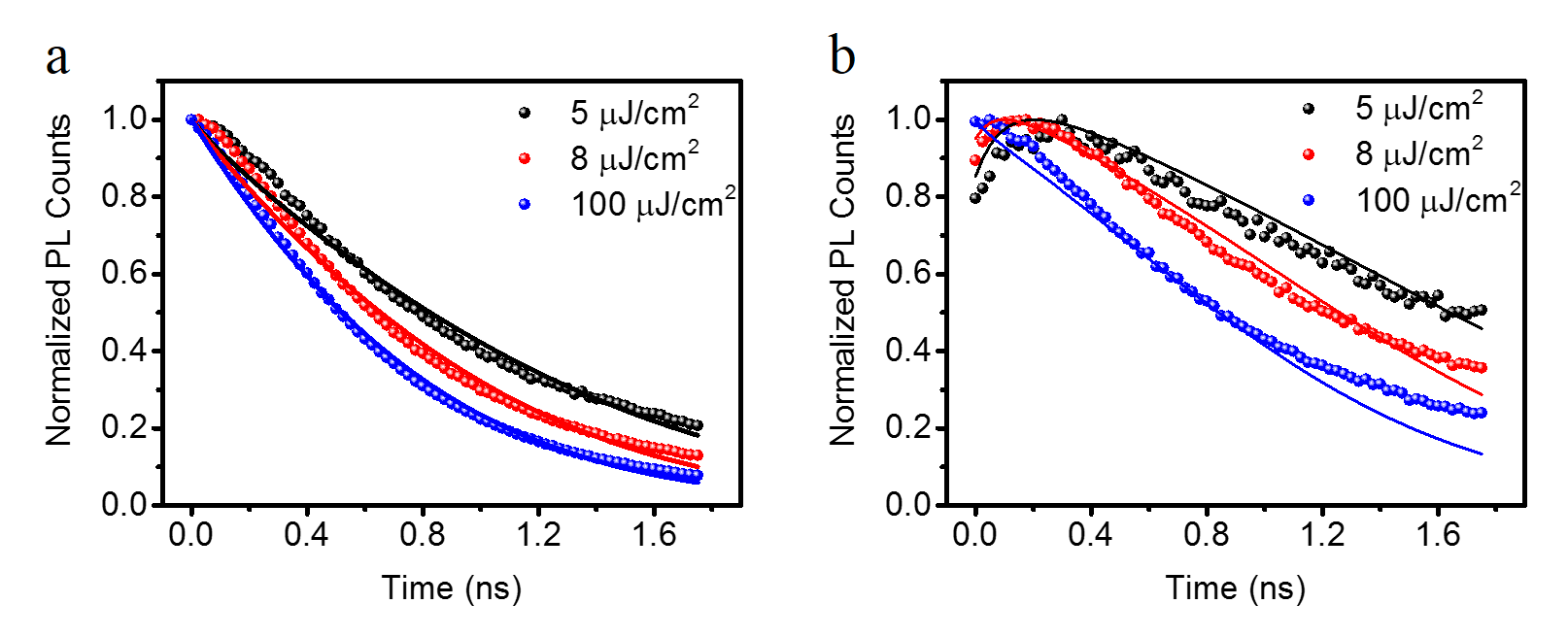
**

**Figure S5. Fluence-dependent TRPL fitting of the MAPbI_3_ thin film at 77 K.** (a) Normalized experimental TRPL data of the HE state (dots) under different excitation fluences. Solid lines are the fittings from the model. (b) Fluence-dependent TRPL of the LE state. The dots are experimental data, and the solid lines are the fitting results.

**Section S4. Temperature-dependent PL and TRPL**

We performed the PL and TRPL measurements as a function of temperature (as shown in Fig. S6a). At low temperature (< 150 K), the MAPbI_3_ film is in the orthorhombic phase and exhibits dual emission peaks, and the peak positions blueshift as the temperature increases. As the temperature exceeds 150 K, the MAPbI_3_ film is in the orthorhombic is in tetragonal phase and only a single peak was observed in the PL spectra. We also investigated the dynamic processes of each emission peak through TRPL measurements (Fig. S6b-d). In Fig. S6d, the charge carriers have a longer lifetime at 50 K than that at 13 K, confirming the communication between the HE state and LE state in orthorhombic phase is a thermal activation process.


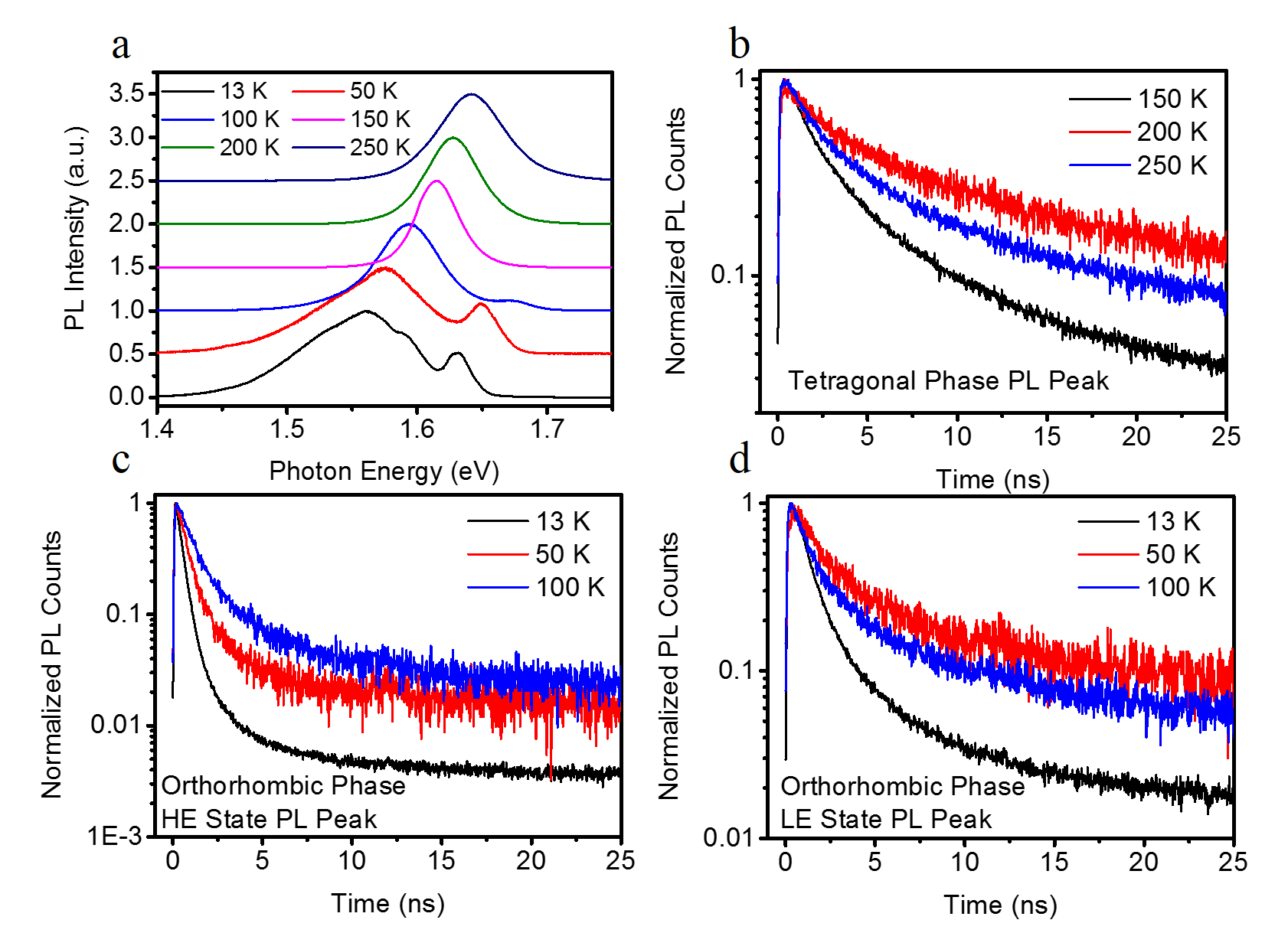


**Figure S6. Temperature dependence of the PL spectra.** (a) Normalized PL intensity of MAPbI_3_ as a function of temperature recorded from 13 K to 250 K, excited by a 532 nm pulsed laser with a fluence of 4 µJ/cm^2^ (spectra have been vertically shifted for clarity). (b) Normalized TRPL of the tetragonal phase emission peak as a function of temperature. (c) Normalized TRPL of the high energy (HE) state in orthorhombic phase from 13 K to 100 K. (d) Normalized TRPL of the low energy (LE) state in orthorhombic phase from 13 K to 100 K.

**Section S5. Spectral-dependent TRPL at room temperature**

Due to the thermal broadening at room temperature, only one PL peak was observed. However, we have performed TRPL spectra at different PL wavelength (emission photon energy) and the results can be shown in Fig. S6. It is evident that at lower emission photon energy (1.59 eV, or 780 nm), we observe the rising feature in the TRPL spectra, different from that of the band edge transition at 1.72 eV (720 nm) but similar to what we have seen from the low energy (LE) state. This confirms that the LE state exists at room temperature, while the thermal broadening renders the distinguished PL peak not spectrally resolved.


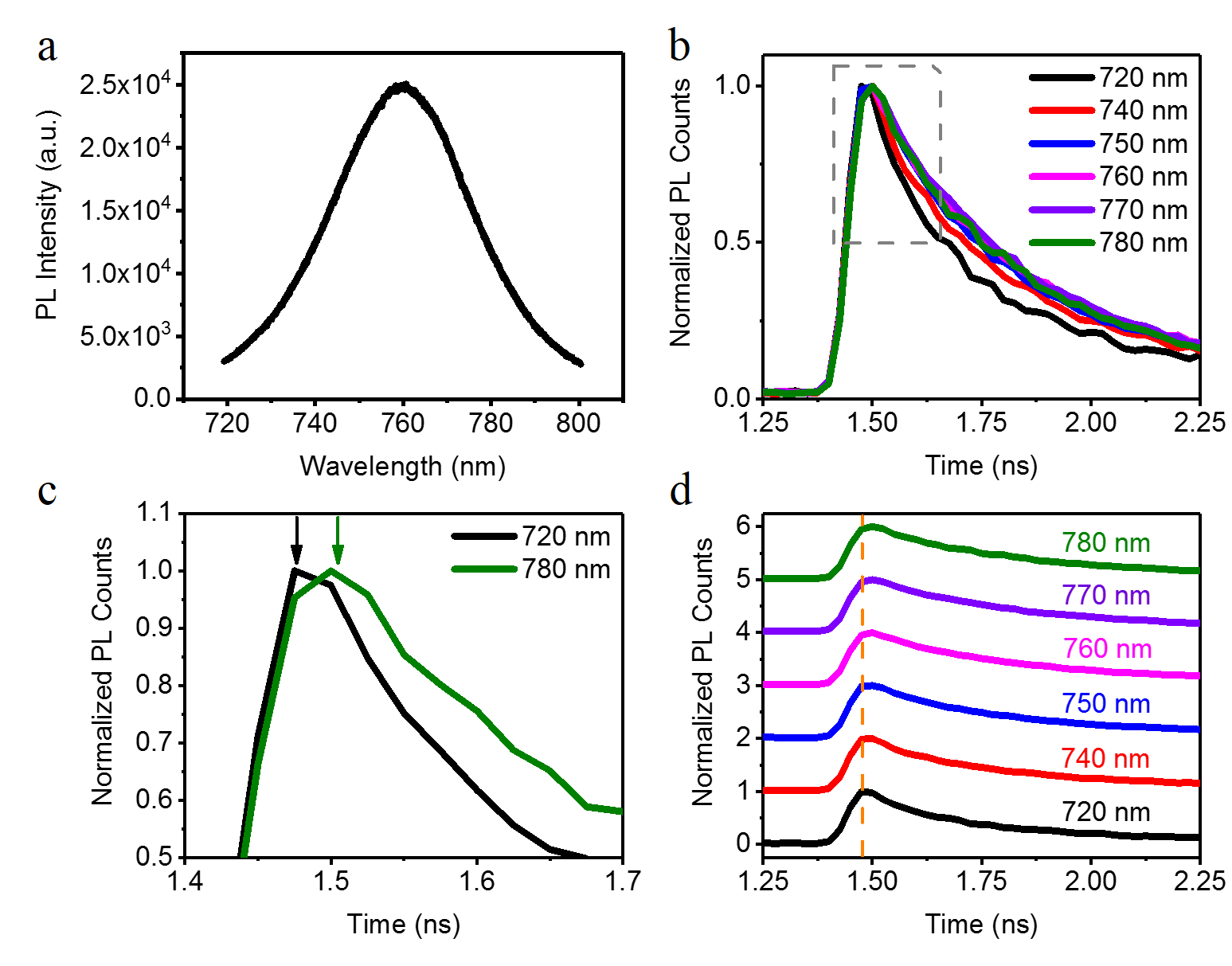


**Figure S7 Excitation wavelength dependent TRPL of MAPBI3 thin film at room temperature.** (a) PL spectra at room temperature with a pulsed laser excitation centered at 2.61 eV (λ = 475 nm). The excitation fluence is 24 µJ/cm2. (b) Normalized TRPL detected at different wavelength. The TRPL at lower wavelength shows a rising feature and longer PL lifetime. (c) A zoom of the TRPL detected at 720 nm and 780nm is shown, highlighting the rising feature at low wavelength.
